# Study on the biomechanical responses of the loaded bone in macroscale and mesoscale by multiscale poroelastic FE analysis

**DOI:** 10.1186/s12938-019-0741-3

**Published:** 2019-12-23

**Authors:** WeiLun Yu, XiaoGang Wu, HaiPeng Cen, Yuan Guo, ChaoXin Li, YanQin Wang, YiXian Qin, WeiYi Chen

**Affiliations:** 1 College of Biomedical Engineering, Shanxi Key Lab. of Material Strength, College of Biomedical Engineering & Structural Impact, Taiyuan University of Technology, Taiyuan, 030024 Shanxi China; 2 Biological Science and Medical Engineering, Beihang University, Beijing, China; 3 Orthopaedic Bioengineering Research Laboratory, Department of Biomedical Engineering, Stony Brook University, Stony Brook, NY USA

**Keywords:** Mechanotransduction, Multiscale, Finite element model, Poroelastic, Biomechanical responses

## Abstract

**Background:**

Bone is a hierarchically structured composite material, and different hierarchical levels exhibit diverse material properties and functions. The stress and strain distribution and fluid flow in bone play an important role in the realization of mechanotransduction and bone remodeling.

**Methods:**

To investigate the mechanotransduction and fluid behaviors in loaded bone, a multiscale method was developed. Based on poroelastic theory, we established the theoretical and FE model of a segment bone to provide basis for researching more complex bone model. The COMSOL Multiphysics software was used to establish different scales of bone models, and the properties of mechanical and fluid behaviors in each scale were investigated.

**Results:**

FE results correlated very well with analytical in macroscopic scale, and the results for the mesoscopic models were about less than 2% different compared to that in the macro–mesoscale models, verifying the correctness of the modeling. In macro–mesoscale, results demonstrated that variations in fluid pressure (FP), fluid velocity (FV), von Mises stress (VMS), and maximum principal strain (MPS) in the position of endosteum, periosteum, osteon, and interstitial bone and these variations can be considerable (up to 10, 8, 4 and 3.5 times difference in maximum FP, FV, VMS, and MPS between the highest and the lowest regions, respectively). With the changing of Young’s modulus (*E*) in each osteon lamella, the strain and stress concentration occurred in different positions and given rise to microscale spatial variations in the fluid pressure field. The heterogeneous distribution of lacunar–canalicular permeability (*k*_lcp_) in each osteon lamella had various influence on the FP and FV, but had little effect on VMS and MPS.

**Conclusion:**

Based on the idealized model presented in this article, the presence of endosteum and periosteum has an important influence on the fluid flow in bone. With the hypothetical parameter values in osteon lamellae, the bone material parameters have effect on the propagation of stress and fluid flow in bone. The model can also incorporate alternative material parameters obtained from different individuals. The suggested method is expected to provide dependable biological information for better understanding the bone mechanotransduction and signal transduction.

## Background

Bone has composite hierarchical structures to achieve diverse mechanical, biological, and chemical functions, such as support and protection, transport, storage cells, and mineral ion homeostasis [1]. The properties of bone are mainly due to its specific hierarchical structure and composition, affording bone characteristics of rigidity, strength, permeability, porosity, toughness, and flexibility, and they can keep close communication and coordination with each other to achieve unified macroscopic functions [1, 2]. The stress and strain distribution and fluid flow in bone play an important role in the effective realization of various functions of bone. Bone structure mainly includes geometry structure, micro-architecture, and material composition. From the scale point of view, it is composed of macroscopic bone tissues, mesoscopic Haversian systems, microscopic lacuna–canalicular system, and nanoscopic collagen fibers and elementary constituents. From the compositional point of view,it is composed of solid phase (type-I collagen and hydroxyapatite crystals) and fluid phase (water and other organic fluids [3]. According to Wolff`s law, bone strength is determined by its structure, and bone structure is regulated by external mechanical stimuli and intelligently adapt to mechanical environment so as to bear the load in the optimum structural form and to obtain the maximum structural strength with the least structural material [4].Within the bone tissue, mechanical stress and bone structure keep a balance relationship that defined the activity of osteoblasts and osteoclasts, and there is a threshold for mechanical stress [5]. Osteoblasts are more active if mechanical stress beyond the maximum threshold, or osteoclasts are more active if the mechanical stress below the minimum threshold [4–7]. Therefore, the distribution of stress and strain in bone affects the bone microstructure and the activity of bone cells. Fluid flow in osteons can produce a series of effect, such as fluid shear stress, pore pressure gradient, solute transport, and streaming potential, and some of these effects can be sensed by osteocytes as signals to trigger bone formation and bone resorption to adapt the continuous change of the mechanical environment [8]. The bone will produce deformation which induced fluid flow in bone under physiological loading, and osteocytes are sensitive to fluid flow and its induced effects [9]. It is significant to research the stress and strain field and the behaviors of fluid flow in different scales of bone under physiological load.

Multiscale model of bone can alternative material parameter of any hierarchical level to determine its effect on bone properties, such as porosity, elastic modulus [3], and permeability [10]. A scaffold composed of materials with multiscale porosity can be used to direct bone regeneration and morphology by controlling the hierarchical structure of the scaffold [11]. Macro-, meso-, and microscale of femoral model were constructed, and the strain amplification factor was found at the lacuna [12]. Pastrama et al. analyzed the influence of pore pressure on bone remodeling, and the pore pressure was caused by the physiological load and transferred from macroscale to microscale [13]. However, a sensitivity analysis of the poroelastic properties of hierarchical structure of bone and a detailed study on the propagation of physiological loading and fluid flow across the length scales of different functional units has not been made yet.

**Fig. 1:**
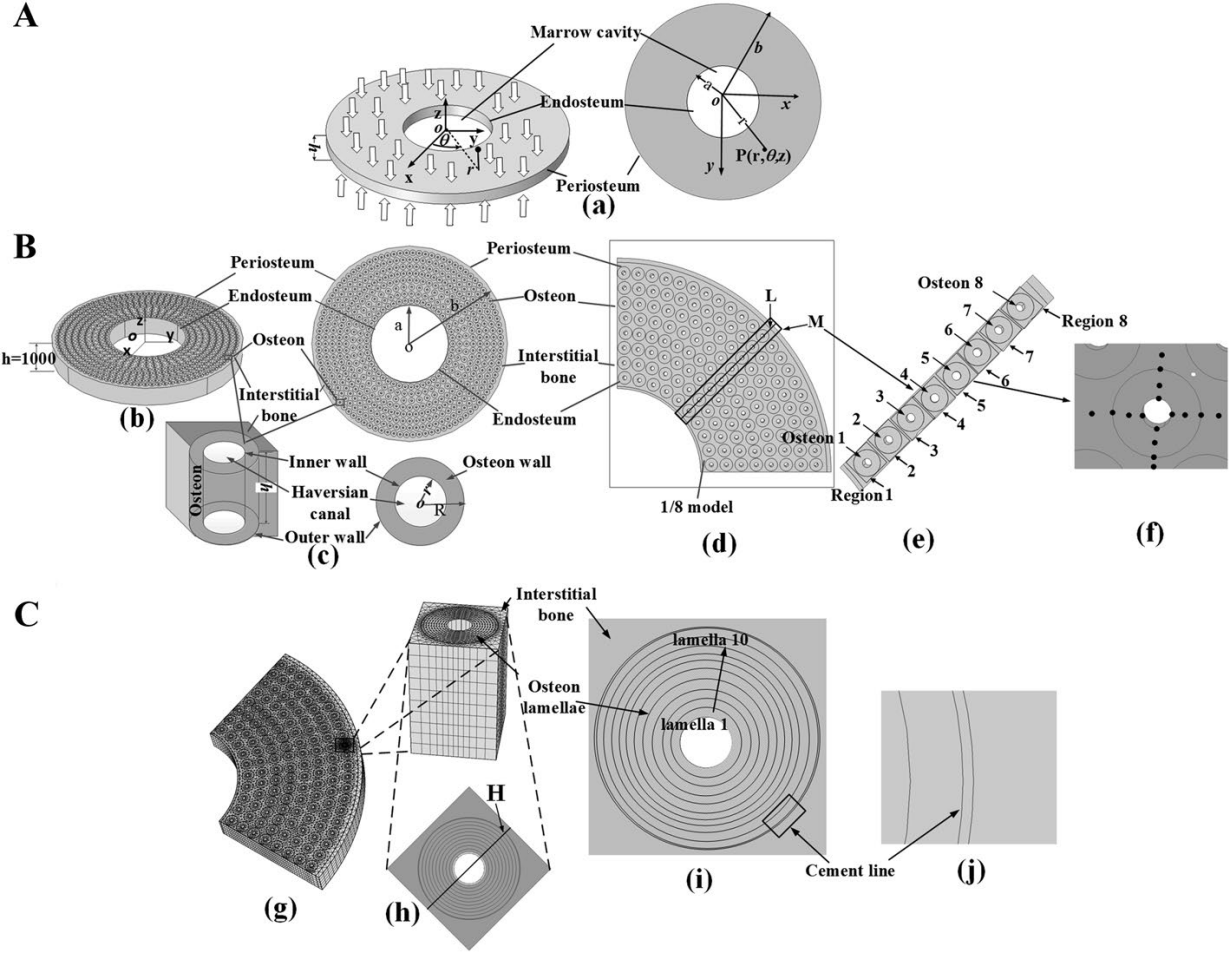
**A** Macroscopic FE model. **a** Macroscopic FE model of segment bone under axial compressive cyclic loading. **B** Macro–mesoscopic model. **b** Macro–mesoscopic model including endosteum, periosteum, interstitial bone, and hollow osteon. **c** The hollow osteon in macro–mesoscopic model. **d** One-eight symmetry model. Regional M and line L are selected to analyze the biomechanical responses along the radius of bone tissue. **e** Region M is divided to 8 small regions (Regions 1–8), and each small region contains one osteon (Osteon 1–8). **f** Some points are selected at the internal wall, the middle and the external wall, and the interstitial bone. **C** Mesoscopic model. **g** FE mesoscopic model including macro–meso interface (including osteon lamellae structure). The critical part near the periosteum in macro–mesoscopic model is refinement. **h** A cube (340 µm) that cut from the macro–mesoscopic model near the periosteum as mesoscale model. Line H is a part of L along radius direction of done tissue. **i** Transversal cross-section of the mesoscale model with 10 osteon lamellae. **j** The cement line

The objective of this research was to develop a multiscale model that included various functional units at each hierarchical level to evaluate the response of poroelastic behaviors under axial compressive cyclic loading. FE analysis is performed three times, at macroscale, macro–mesoscale, and mesoscale structural levels (Fig. 1, Tables 1, 2, 3 and 4), with each analysis at a greater level of refinement, using COMSOL Multiphysics software. This model neglected the bone marrow cavity and trabeculae, only considers the tissue from the endosteum to periosteum, and the mechanical property and the flowing discipline of various functional units was observed. At the mesoscale, an osteon which cut from the whole model was refined, and the effects of *E* and *k*_lcp_ on the fluid flow and stress and strain field were investigated. All properties and parameters were based on literature reports, and the solid structure and interstitial fluid were assumed as transverse isotropic poroelastic material and compressible liquid, respectively. This paper provides a deeper understanding of the mechanotransduction and stimulation by fluid flow which induces bone remodeling and bone metabolism.

**Table 1:** Material constants used in macroscale model

Parameter	Description	Value
*E*_*r*_	Radial drained Young’s modulus	14.58 (GPa)
*ν*_*r*_	Radial drained Poisson’s ratio	0.325
*E*_*z*_	Axial drained Young’s modulus	20.3 (GPa)
*ν*_*z*_	Axial drained Poisson’s ratio	0.25
M	Biot’s modulus	38 (GPa)
α	Biot’s effective coefficient	0.12
*ϕ*_*v*_	Vascular porosity	0.04
*ρ*_S_	Solid density	2000 (kg/m^3^)
*ρ*_f_	Fluid density	1000 (kg/m^3^)
*k*_vp_	Vascular permeability	10^−15^ (m^2^)
µ	Dynamic viscosity	10^−3^ (Pa s)
a	Inner radius of bone tissue	2 (mm)
b	Outer radius of bone tissue	5 (mm)
*C*_*p*_	Fluid compressibility	4 × 10^−10^ (1/Pa)

**Table 2:** Material parameters used in the macro–mesoscopic FE model

	Osteon	Interstitial bone	Endosteum	Periosteum
*E*_*r*_	15.75 (GPa)	17.325 (GPa)	4.41(MPa)	4.41(MPa)
*v*_*r*_	0.328	0.2925	0.49	0.49
*E*_*z*_	20.3(GPa)	22.33(GPa)	25.67(MPa)	25.67(MPa)
*v*_*r*_	0.25	0.225	0.49	0.49
*k*_lcp_	10^−19^ m^2^	10^−19^ m^2^	2.7 × 10^−16^ m^2^	2.7 × 10^−16^ m^2^

**Table 3:** Settings of osteon lamella *E* for poroelastic analysis (GPa)

Case 1	*E*_*r*_1, E_z_1	*E*_*r*_2, E_z_2	*E*_*r*_3, E_z_3	*E*_*r*_4, *E*_*z*_4	*E*_*r*_5, *E*_*z*_5
	11, 14.3	12, 15.6	13, 16.9	14, 18.2	15, 19.5
	*E*_*r*_6, E_z_6	*E*_*r*_7, E_z_7	*E*_*r*_8, E_z_8	*E*_*r*_9, *E*_*z*_9	*E*_*r*_10, *E*_*z*_10
	16,20.8	17, 22.1	18, 23.4	19, 24.7	20, 26
Case 2	*E*_*r*_1, E_z_1	*E*_*r*_2, E_z_2	*E*_*r*_3, E_z_3	*E*_*r*_4, *E*_*z*_4	*E*_*r*_5, *E*_*z*_5
	20, 26	19, 24.7	18, 23.4	17, 22.1	16, 20.8
	*E*_*r*_6, E_z_6	*E*_*r*_7, E_z_7	*E*_*r*_8, E_z_8	*E*_*r*_9, *E*_*z*_9	*E*_*r*_10, *E*_*z*_10
	15, 19.5	14, 18.2	13, 16.9	12, 15.6	11, 14.3
Case 3	*E*_*r*_1, E_z_1	*E*_*r*_2, E_z_2	*E*_*r*_3, E_z_3	*E*_*r*_4, *E*_*z*_4	*E*_*r*_5, *E*_*z*_5
	20, 26	18, 23.4	16, 20.8	14, 18.2	12, 15.6
	*E*_*r*_6, E_z_6	*E*_*r*_7, E_z_7	*E*_*r*_8, E_z_8	*E*_*r*_9, *E*_*z*_9	*E*_*r*_10, *E*_*z*_10
	12, 15.6	14, 18.2	16, 20.8	18, 23.4	20, 26

**Table 4:** Settings of osteon lamella permeability (*k*_lcp_) for poroelastic analysis (m^2^)

Case 4	*k*_lcp_1	*k*_lcp_2	*k*_lcp_3	*k*_lcp_4	*k*_lcp_5
	23 × 10^−19^	21 × 10^−19^	19 × 10^−19^	17 × 10^−19^	15 × 10^−19^
	*k*_lcp_6	*k*_lcp_7	*k*_lcp_8	*k*_lcp_9	*k*_lcp_10
	13 × 10^−19^	11 × 10^−19^	9 × 10^−19^	7 × 10^−19^	5 × 10^−19^
Case 5	*E*_*r*_1, E_z_1	*E*_*r*_2, E_z_2	*E*_*r*_3, E_z_3	*E*_*r*_4, *E*_*z*_4	*E*_*r*_5, *E*_*z*_5
	5 × 10^−19^	7 × 10^−19^	9 × 10^−19^	11 × 10^−19^	13 × 10^−19^
	*k*_lcp_6	*k*_lcp_7	*k*_lcp_8	*k*_lcp_9	*k*_lcp_10
	15 × 10^−19^	17 × 10^−19^	19 × 10^−19^	21 × 10^−19^	23 × 10^−19^
Case 6	*k*_lcp_1	*k*_lcp_2	*k*_lcp_3	*k*_lcp_4	*k*_lcp_5
	23 × 10^−19^	21 × 10^−19^	19 × 10^−19^	17 × 10^−19^	15 × 10^−19^
	*k*_lcp_6	*k*_lcp_7	*k*_lcp_8	*k*_lcp_9	*k*_lcp_10
	15 × 10^−19^	17 × 10^−19^	19 × 10^−19^	21 × 10^−19^	23 × 10^−19^

## Results

### Comparison of finite element method and numerical simulation

**Fig. 2:**
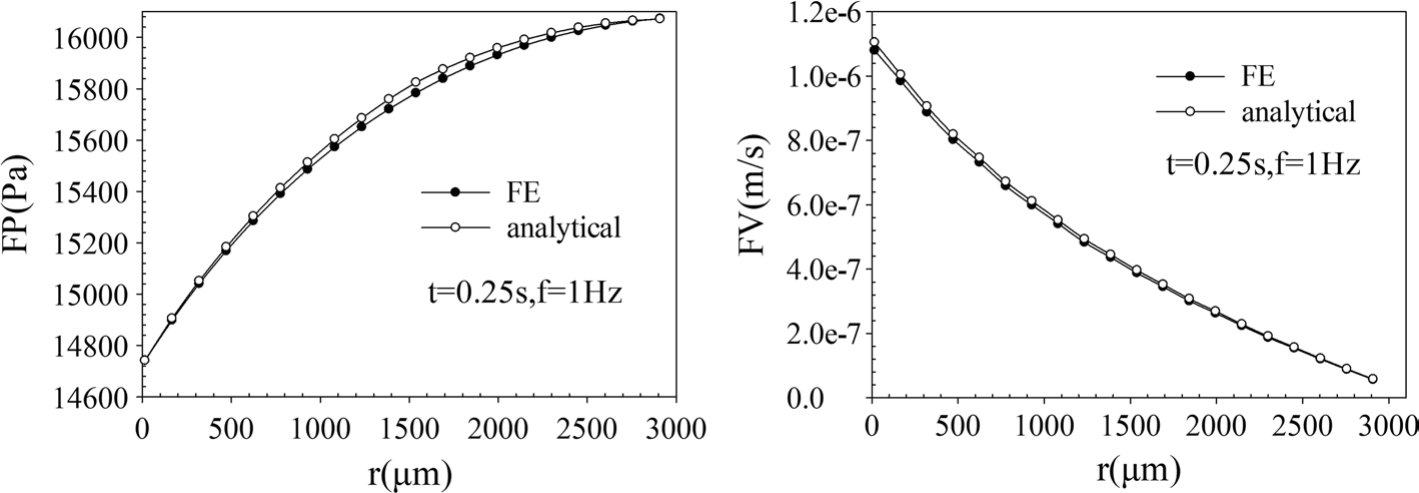
Comparison of the analytical data and FE data of bone model at *t* = 0.25 s. Fluid pressure FP (left) and velocity FV (right) vs bone radius

The comparison of FP and FV along the radial direction of macroscale model (Fig. 1A) was shown in Fig. 2, and the computed results of the macroscale FE model showed good agreement with the results of numerical simulation, and the result error was acceptable, which verified the validity of the model.

### The analyses of macro–mesoscopic FE model

**Fig. 3:**
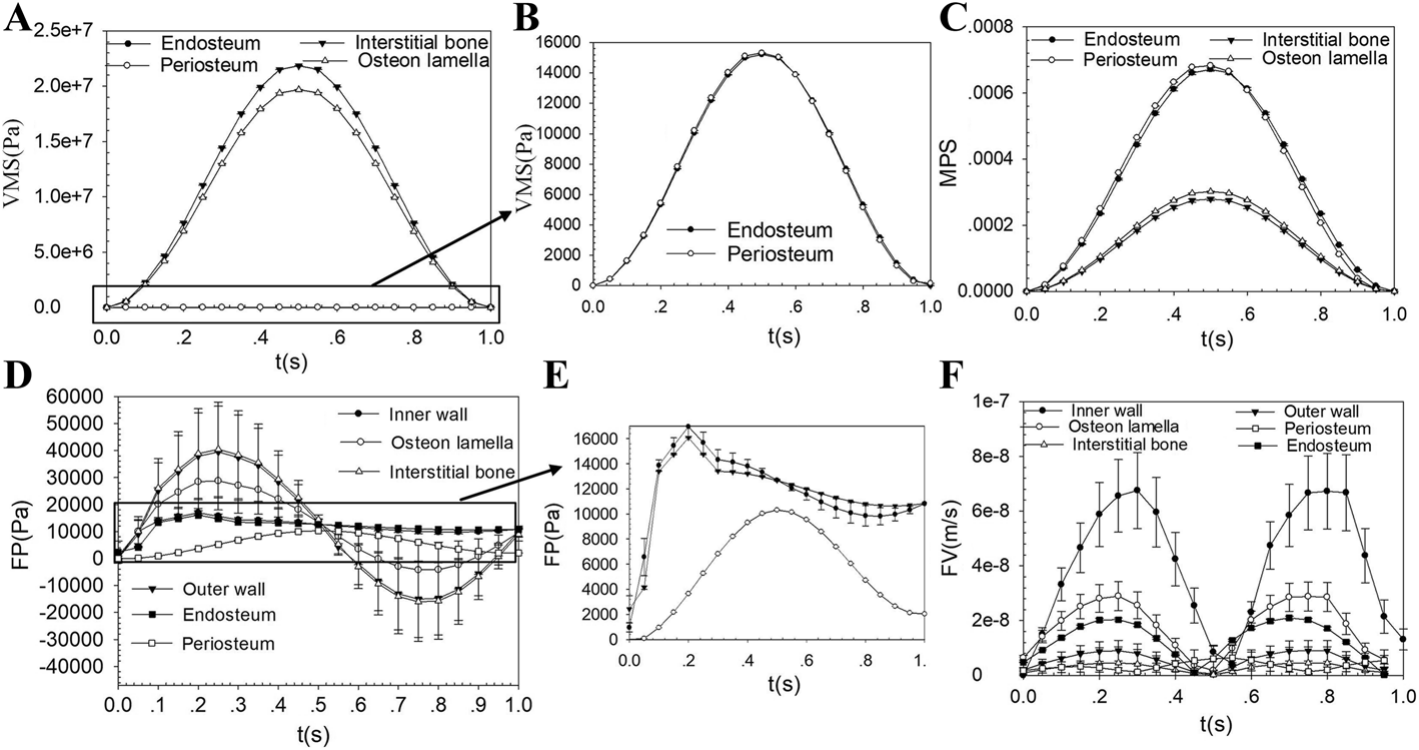
Detailed analyses of the biomechanical responses of selected locations in Fig. 1f versus loading time. **A** The mean VMS of different locations versus time. **B** The enlarged region of part of (**A**). **C** The mean MPS of different locations versus time. **D** The mean FP of different locations versus time. **E** The enlarged region of part of (**D**). **F** The mean FV of different locations versus time

Because of the symmetry of geometric structure (Fig. 1B, C), in order to reduce the computation, 1/8 model was established (Fig. 1d). A region M was taken along the radius of the bone tissue (Fig. 1d), and some points were get at different positions of the endosteum, interstitial bone, osteon, and periosteum within the region M. The mean values of VMS (Fig. 3A, B), MPS (Fig. 3C), FP (Fig. 3D, E), and FV (Fig. 3F) with the loading time at these points were calculated, and we found that the VMS and MPS reached maximum values at around *t* = 0.5 s, and the FP and FV reached maximum values at around *t* = 0.25 s. At *t* = 0.5 s, average VMS (± standard deviation) from endosteum, osteon, interstitial bone, and periosteum regions was about 6.89 × 10^6^ ± 3.81 × 10^6^ Pa, 2.04 × 10^7^ ± 5.27 × 10^5^ Pa, 2.04 × 10^7^ ± 2.80 × 10^6^ Pa, and 6.20 × 10^6^ ± 3.44 × 10^6^ Pa, respectively, and average MPS was about 7.56 × 10^−4^ ± 7.99 × 10^−5^, 2.69 × 10^−4^ ± 2.72 × 10^−6^, 2.5 × 10^−4^ ± 7.17 × 10^−5^, and 6.65 × 10^−4^ ± 9.83 × 10^−5^, respectively. At *t* = 0.25 s, average FP was about 14,789 ± 3 Pa, 32,241 ± 10,700 Pa, 42,538 ± 14,533 Pa, and 5263 ± 2.24 Pa, respectively, and average FV was about 4.05 × 10^−8^ ± 8.4 × 10^−9^ m/s, 3.45 × 10^−8^ ± 1.91 × 10^−8^ m/s, 1.12 × 10^−8^ ± 6.58 × 10^−9^ m/s, and 1.72 × 10^−8^ ± 1.14 × 10^−8^ m/s. It was worth noting that the FV was the absolute value of velocity.

**Fig. 4:**
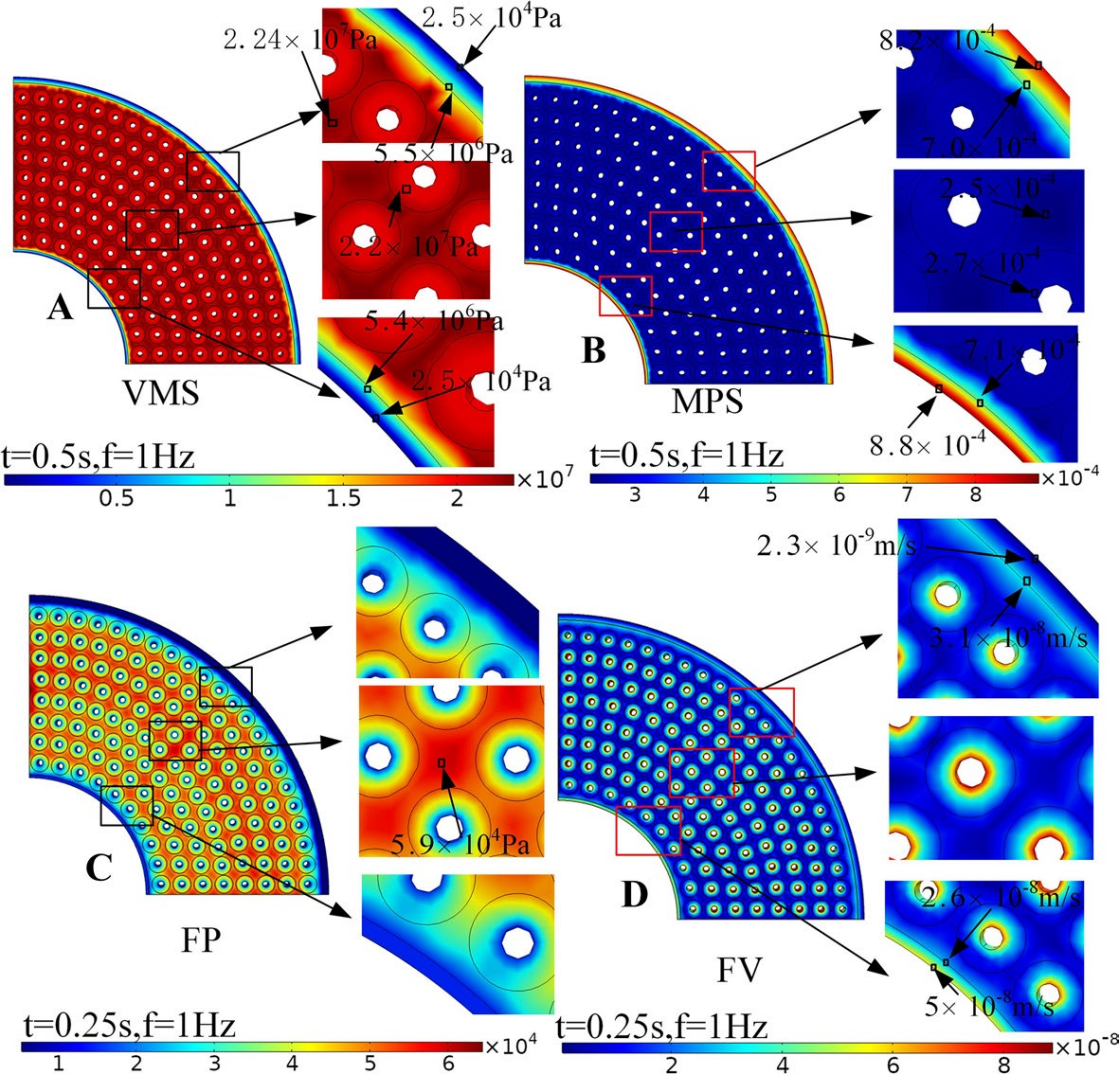
The distributions of VMS (**A**), MPS (**B**), FP (**C**), and FV (**D**) under axial compressive cyclic loading

The distributions of the maximum response of VMS, MPS, FP, and FV were shown in Fig. 4. The maximum VMS at interstitial bone was 2.24 × 10^7^ Pa, which was generally higher than that in other locations (Fig. 4A). The maximum VMS at periosteum (5.5 × 10^6^ Pa) and endosteum (2.5 × 10^5^ Pa) had minimum values among all functional units (Fig. 4A), while the maximum MPS (8.4 × 10^−4^ and 8.8 × 10^−4^) was significantly higher than other locations (Fig. 4B). The maximum FP around the endosteum (14,786 Pa) and periosteum (5264 Pa) was significantly lower than other locations (Fig. 4C). In the same small region of bone tissue, the maximum FP of interstitial bone (5.9 × 10^4^ Pa) was greater than osteon (4.8 × 10^4^ Pa) (Fig. 4C), but the FV of interstitial tissue (1 × 10^−8^ m/s) was less than osteon (8 × 10^−8^ m/s) (Fig. 4D). The FV in the area where the periosteum and bone tissue contact had significant changes (Fig. 4D).

### The analyses of osteon level in macro–mesoscopic FE model

**Fig. 5:**
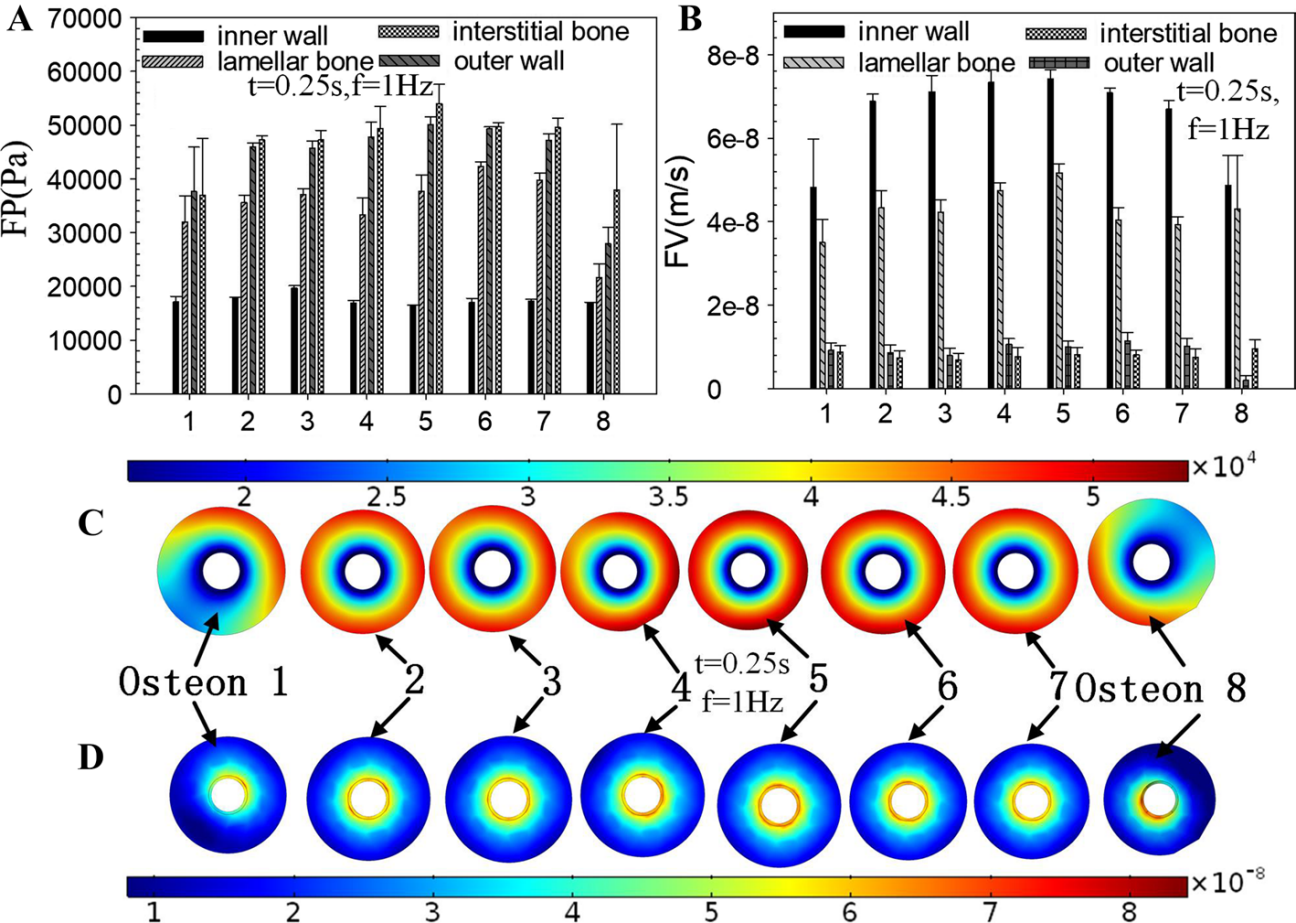
Detailed analyses of the maximum response of FP (**A**) and FV (**B**) at different locations in Region 1–Region 8 (Fig. 1**e**). The distributions of FP (**C**) and FV (**D**) of Osteon 1–Osteon 8 (Fig. 1**e**)

Some points were selected at the internal wall, the middle, the external wall, and the interstitial bone in Region 1–Region 8 (Fig. 1e, f) to analyze and compared the response of FP and FV, respectively. The peak values of Region 2–Region 7 have no significant difference; however, the peak values of Region 1 and Region 8 were significantly smaller than other regions. The peak FP decreases gradually from interstitial bone to the inner wall of osteon (Fig. 5A). The FV at the inner wall of osteon had the maximum value, and the range of peak FV in osteon was about 5 × 10^−8^m/s–8 × 10^−8^m/s (Fig. 5B). The value of FV in both interstitial bone and outer wall of osteon was less than 2 × 10^−8^m/s (Fig. 5B), which meant that the fluid stimulation generated by fluid flow in these locations was too small to cause the response of osteocyte’s mechanoreceptors and was called “dead zone” [14].

The FP (Fig. 5C) and FV (Fig. 5D) of Osteon 2–7 showed the symmetrical distribution around the Haversian canal, but because of the influences of the boundary conditions, the FP and FV of Osteon 1 and Osteon 8 were asymmetric distribution. The FP and FV on the side near endosteum and periosteum both had a trend of decreasing markedly.

### Effects of Young’s modulus (*E*) and permeability (*k*_lcp_) on mesoscale FE model

**Fig. 6:**
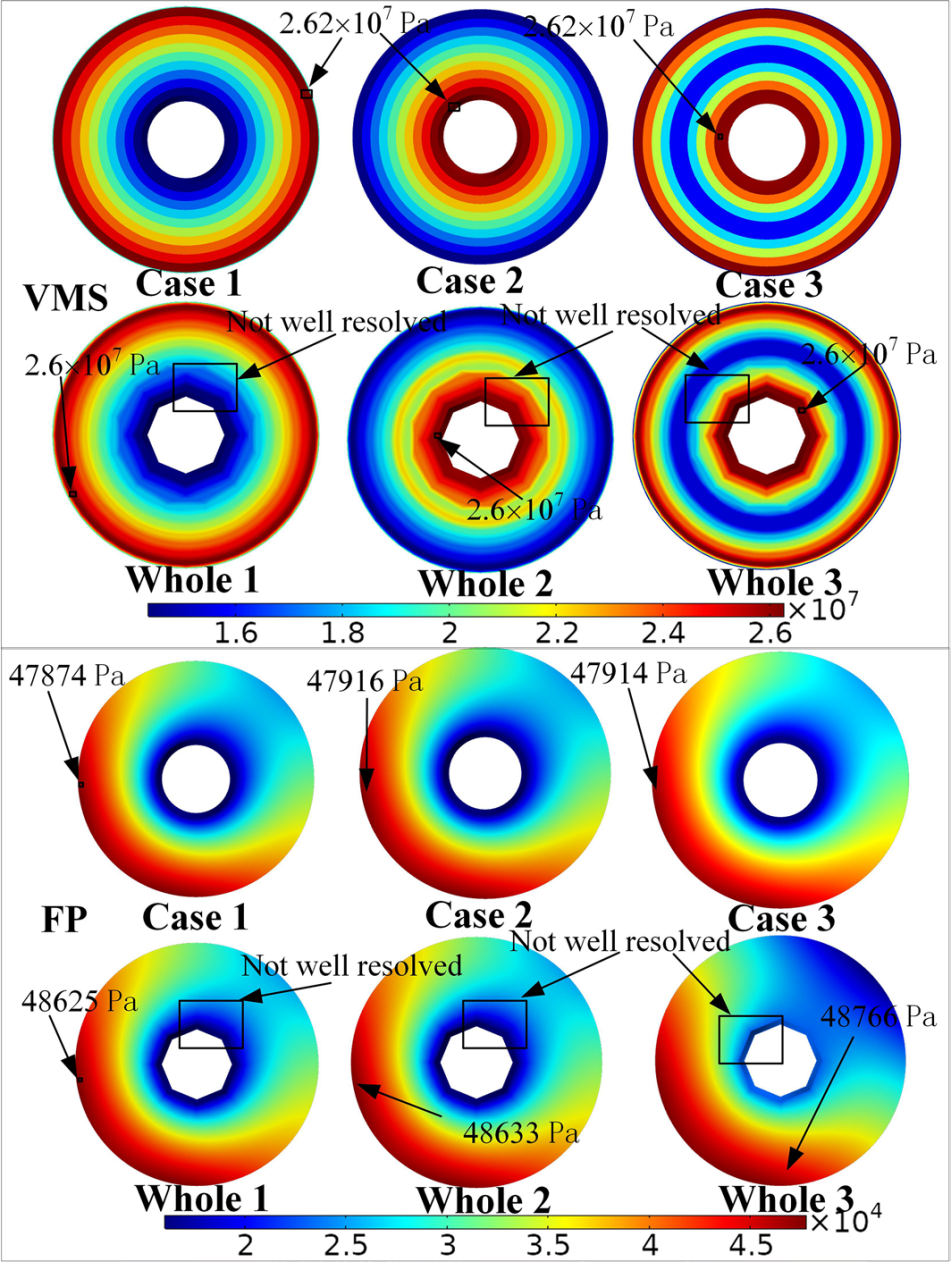
The distributions of maximum VMS and FP over the selected osteon. The whole model (Whole 1, 2, and 3) and the mesoscale models (Cases 1, 2, and 3) are compared

As a verification, the VMP and FP states at the cuts where the mesoscale models had displacements and pressure prescribed by the results in the whole model (Fig. 1g) were checked. Such a comparison was shown in Fig. 6. For the range of parameters investigated, the computed peak VMS of the whole macro–mesoscopic models (Whole 1, 2, and 3) and mesoscale models (Case 1, 2, and 3) was about 2.6 × 10^7^ Pa and 2.62 × 10^7^ Pa, respectively, and the peak value of FP was about 4.8 × 10^4^ Pa and 4.7 × 10^4^ Pa, respectively. The peak values of VMS and FP for the whole models were about 0.1% ~ 1% and 1% ~ 2% different compared to that in the mesoscale models, which were expected given the coarse mesh in the whole model. The results strongly indicate that the mesoscale models had been set up correctly. The cut through the whole models (Whole 1, 2, and 3) in Fig. 6 displayed that the stresses and pressures were not well resolved. The stress and pressure fields are not smooth, especially around Haversian canal. Due to the large size span of the whole model, sometimes it is not feasible to have a mesh that at the same time captures the global behavior and resolves the detail structures with high accuracy. For thin bone lamella structures, it is common that the regions with different properties are small. The mesoscale models can simulate the detail structures more accurately. In the corresponding figures from the mesoscale models, Case 1, Case 2, and Case 3, the stress and pressure field were smooth and well resolved. The finer mesh in the mesoscale models was better than the coarser mesh of the whole models in capturing the changes of VMP, MPS, FP, and FV. It was, however, observed in real physiological environment that a thin layer was required. If the bone lamellae were to be created in all osteons of the whole model, the simulation would require large computational resources. A Free Tetrahedral mesh was used. The whole model with mesoscopic structure (which is only in the selected osteon) consists of 58,180 elements and the mesoscale model consists of 33,256 elements. To better resolve details with the mesh, we set maximum element size 500 μm, minimum element size 5 μm, curvature factor 1.5, resolution of narrow regions 0.6 and maximum element growth rate 0.5 in the whole model, and set maximum element size 27.5 μm, minimum element size 2 μm, curvature factor 1.4, resolution of narrow regions 0.4 and maximum element growth rate 0.7 in the mesoscale model. In the current example, the whole model consisted of about 1,101,293 degree of freedoms while the mesoscale models consisted of 245,221 degree of freedoms.

**Fig. 7:**
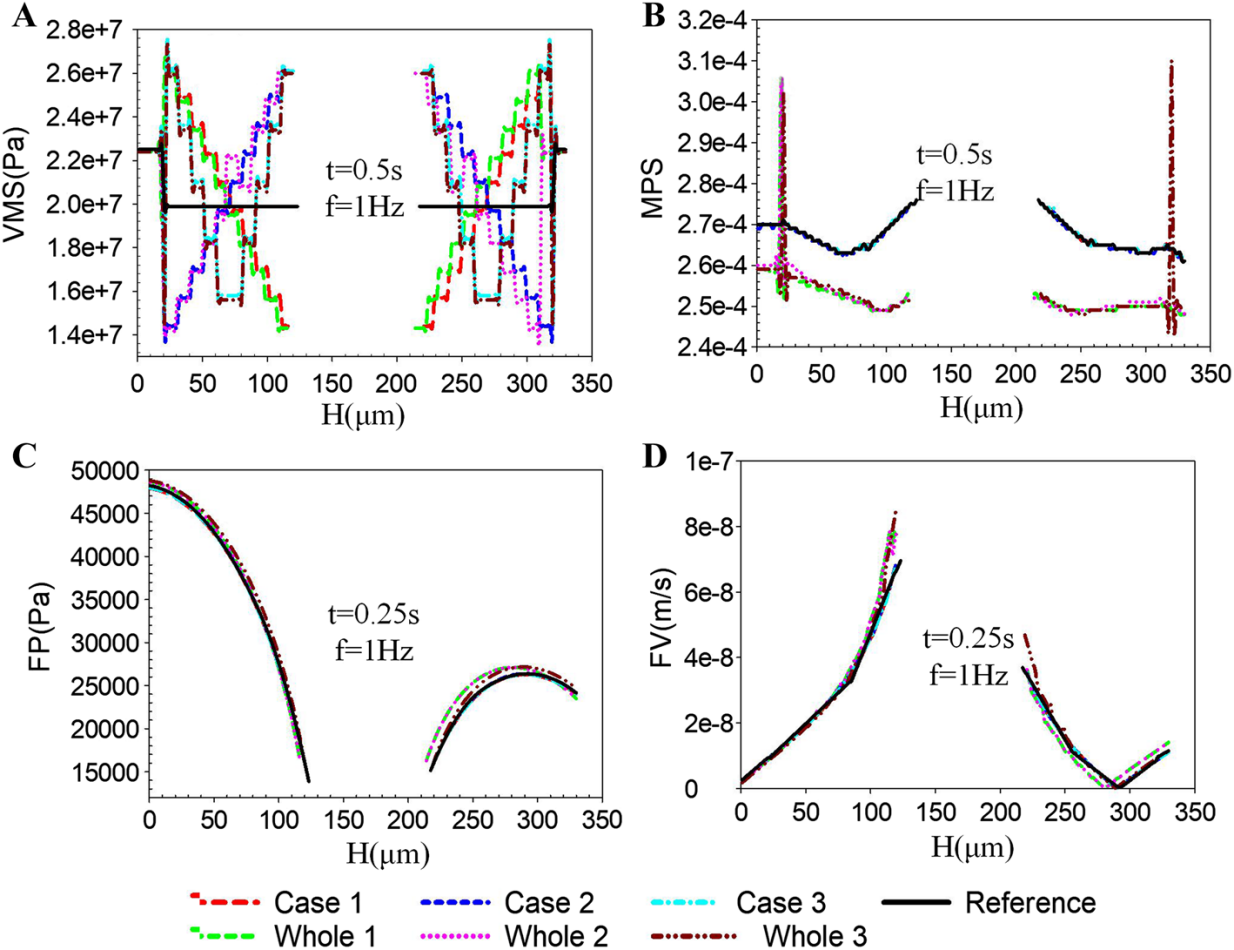
Effects of different *E* in each osteon lamella on mesoscale model. The maximum responses of VMS (**A**), MPS (**B**), FP (**C**), and FV (**D**) along the line H for the whole models (Whole 1, 2, and 3) and the mesoscale models (Cases 1, 2, and 3)

In order to investigate the effect of the *E* and *k*_lcp_ of different osteon lamellae on its poroelastic mechanical behavior, a line segment H (Fig. 1h) was selected along the radius direction of the bone tissue passing through the mesoscale model, and the changes of VMS, MPS, FP, and FV on H were observed. The difference between the VMP, MPS, FP, and FV of the whole models and of the mesoscale models was small. The *E* had significantly effect on the VMP (Fig. 7A), MPS (Fig. 7A), FP (Fig. 7A), and FV (Fig. 7A). With the different *E* of each osteon lamella, the VMS of the bone lamella showed gradient change (Fig. 7A). As shown in Fig. 7B, the local MPS at medial lamellae position increased obviously in three cases, and the local MPS in Case 1 was significantly larger than Case 2 and Case 3. As shown in Fig. 7A, B, the peak VMS in Case 1 was at lateral lamellae, while the peak VMS occurred at medial lamellae in Case 2 and Case 3.

**Fig. 8:**
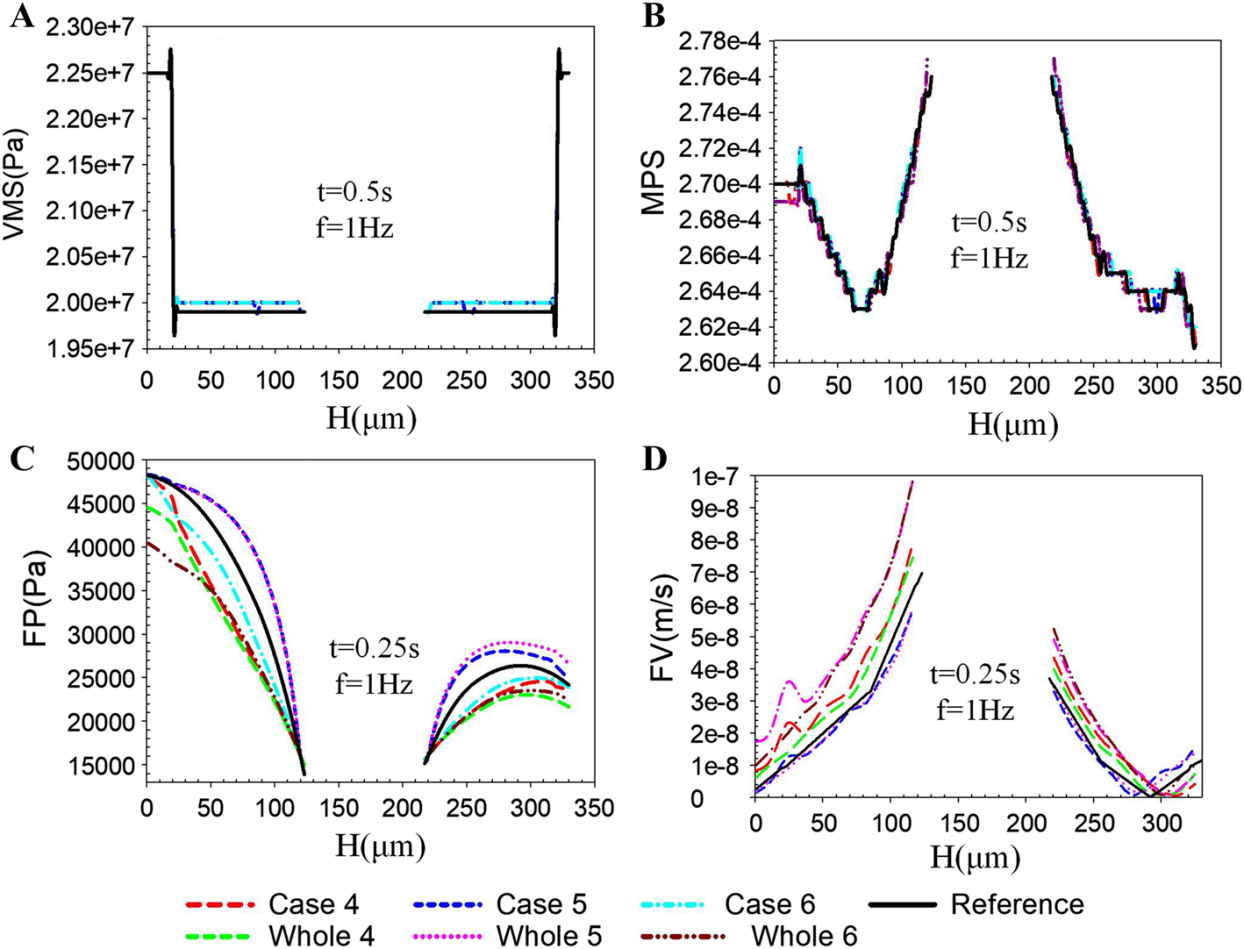
Effects of different *k*_lcp_ in each osteon lamella on mesoscale model. The maximum responses of VMS (**A**), MPS (**B**), FP (**C**), and FV (**D**) along the line H for the whole models (Whole 4, 5, and 6) and the mesoscale models (Cases 4, 5, and 6)

Figure 8 shows that the *k*_lcp_ had significantly effect on the FP (Fig. 8C) and FV (Fig. 8D) of osteon lamellae, but had a little impact on the VMS (Fig. 8A) and MPS (Fig. 8B). As shown in Fig. 8D, due to the low permeability of the interstitial bone and cement line, the FV had an increasing trend in the junction of the cement line and osteon than the reference.

## Discussion

This paper presented a multiscale poroelastic FE model under axial compressive cyclic loading to analyze the stress and strain field and fluid flow of bone. The distributions of the VMS, MPS, FP, and FV in endosteum, interstitial bone, osteon, and periosteum were analyzed on macro–mesoscopic model, and the effects of *E* and *k*_lcp_ on stress and strain field and fluid flow in mesoscopic model were investigated.

The osteogenic function of periosteum is more important than the bone marrow and endosteum, and the periosteum has abundant pluripotent stem cells and molecular factors of regulating cell behavior play an important role in providing cells resource for bone repairing and bone healing [15, 16]. The material properties of periosteum selected in our model, which was considered to have abundant osteoprogenitor cells [17], made it more compliant. This had important effects as periosteum deform elastically at small strains. As shown in Fig. 4B, the MPS on the periosteum and endosteum was much larger than the interstitial bone and osteon, which results in a large strain gradient that may lead to greater strain stimulation on the bone cells on the endosteum and periosteum. However, the use of a transversely isotropic model for the periosteum and endosteum with a small elastic modulus of 4.41 MPa in radial direction and 25.67 MPa in axial direction resulted in a much lower VMS (Fig. 4A). The large VMS (Fig. 4A) in interstitial bone makes it easy to produce crack and fatigue damage, and the properties of high VMS and crack-prone of interstitial bone can provide a protection to the osteon, which helped to protect the osteocyte and maintained normal metabolic activity in the bone. This result was reasonable in the functions of maintaining structural integrity and resistance to fatigue damage in the process of biology evolution.

The maximum FP and FV of the whole bone tissue tend to be in a distribution of great in the middle and small at both ends (Fig. 5). The higher the FP and FV in osteon, the stronger the fluid stimulation is perceived by the osteocytes, which may lead to the physiological activities such as bone remodeling and bone metabolism more active. The distributions of FP and FV of Osteon 2–7 in Fig. 5 had little difference, which suggested that the fluid stimulation that the osteocytes felt in these osteons was basically the same. However, the asymmetrical distributions of FP and FV in Osteon 1 and Osteon 8 were found. The difference of FP and FV in Osteon 1, Osteon 8, and Osteon 2-7 may lead to different mechanosensation in osteocytes. The difference may relate to endosteum and periosteum. Studies had shown that the endosteum and the periosteum had important material exchange and signal transduction with bone tissue [18]. The periosteum and endosteum are closely related to the activities of bone formation and bone resorption, which together maintain the stability of the thickness of cortical bone [19]. The FV at the inner wall of the osteons was much larger than other positions, with a peak value about 8 × 10^−8^m/s (Fig. 5B). The maximum FV in both interstitial bone and outer wall of osteon was less than 2 × 10^−8^m/s (Fig. 5B), which means that the fluid stimulation generated by fluid flow in these locations was too small to cause the response of osteocyte’s mechanoreceptors and was called “dead zone” [14].

The variations of *E* in 3 cases in the mesoscale model are all derived from the experimental observations [20–23]. As shown in Fig. 6, there were obvious high stress at the medial lamella in Case 2 and Case 3. High stress can lead to initiation of cracking by fatigue. Therefore, it is easy to appear microcrack damage near the Haversian canals in Case 2 and Case 3. Under physiological load, the bone formation of osteoblasts interacted with bone resorption of osteoclasts to complete the growth of new bone and the removing of old bone, and therefore, there would be more new bone formations in the large stress region to form strong bone lamellae. According to the geometry of osteon, the redistribution of stress in the existing structure will be hindered by Haversian canal, which should lead to the presence of stress concentration. The existence of stress concentration around the Haversian canal will lead to the increase of *E* (Case 2 and Case 3), which conformed to principle of bone adaptability. In Case 1 (Fig. 6), the lateral bone lamellae had higher stress and medial bone lamellae had lower stress, and the high stress region of lateral bone lamellae could cause stress shielding to medial bone lamellae. This stress shielding effect can prevent the stress concentration around Haversian canal to damage the osteon and provide protection to osteon [24]. Therefore, the results in Case 1 also conformed to the functions of maintaining structural integrity and resistance to fatigue damage in biological evolution. As shown in Fig. 7C, D, the different distributions of *E* had influence on the FP and FV, which indicated that the variations of *E* had effect on the fluid flow of osteon. The microcracks could affect the fluid flow behaviors of osteon [25]. The osteocytes around the Haversian canal could feel larger fluid stimulation (Figs. 5B, 7D and 8D), which may be related to the high stress around the Haversian canal and the susceptibility to initiation of microcracks. The existence of stress concentration and microcracks could make the bone formation more frequent to increase the strength of bone lamellae and repair microcracks.

Permeability can be regarded as a macro-index to describe the microscopic flow behavior in bone tissue [26, 27]. According to previous theoretical and experimental results, the range of *k*_lcp_ was 10^−17^m^2^ to 10^−25^m^2^ [28]. The variations of *k*_lcp_ in bone lamellae might be related to the distribution of the bone canaliculi and lacuna, the low-permeability regions might have less bone canaliculi and lacuna [29]. Although researchers had done extensive research on the permeability of bone, the effect of gradient distribution of permeability of osteon lamellae on the biological responses of osteocyte under fluid flow was still unclear. Our study found that the heterogeneous distribution of permeability in osteon lamella had a significant effect on FP and FV (Fig. 8). The FV was found to fluctuate at bone lamellae and cement line that was different from the findings of [10, 26]. In reality, the micropore of bone tissue may pass through the cement line [30], so the cement line may be permeable. Although the permeability value of the cement line is still unclear, this permeability of the cement line may change the mechanical behavior of cortical tissue. Therefore, we propose that the osteon in the ideal model is covered by a cement line. In addition, because the permeability of the cement line is lower than that of the osteon, the flow velocity at the cement line increases obviously, which will increase the fluid stimulation of the osteocytes near the outer wall of osteon. This mesoscale model was investigated by multiscale method, and the boundary conditions were more approach to real mechanical environment.

This paper established multiscale FE model to study strain and stress distributions and the fluid flow based on the poroelastic theory and provided a foundation for further exploration of the micromechanism of the growth and differentiation of osteocyte. Furthermore, we were restricted to simplified finite element models, whereas the bone tissue has more complex geometric structure, boundary conditions, and physiological load conditions. Incorporation of a more realistic model of bone tissue would prove useful for a more accurate representation of the different responses of stress and strain distribution and fluid flow when under physiological load. Future refinements of the model will address the inherent limitations. Nevertheless, this study represented a significant step in developing a multiscale model of bone tissue that incorporated explicit representation of osteon lamellae and cement line parameters. The future work is to establish microscale model based on the multiscale method and further quantify the load and fluid flow signal transfer from macroscale and mesoscale to microscale.

## Conclusions

In summary, a multiscale poroelastic finite element method for the hierarchical structure of bone tissue was developed under axial compressive cyclic load, and the distribution of stress and strain and fluid flow in bone were investigated in different scales. Multiscale method can reflect the real physiological environment of different layers more accurately than research each component of bone separately. The further model can be used to analyze the effect of bone scaffold, bone substitute or implants on the stress and fluid flow distributions of bone tissue to more accurately assess the potential beneficial and harmful effects, so as to accurately achieve better individual matching. This work provides a better understanding of fluid flow and mechanotransduction in bone remodeling.

## Methods

### Governing equations for poroelastic bone model

Poroelasticity was first developed by Biot and widely used in solid–liquid coupling poroelastic materials [31]. The following governing equations can be used to describe the poroelastic behavior of bone, and no body forces are taken into account [14, 26]. Constitutive laws for the solid matrix material and the saturating fluid can be written as:

1 $${\varvec{\upsigma}} = {\mathbf{M\varepsilon }} - {\varvec{\upalpha}}p$$

2 $$p = M\left[ {\xi - \text{tr} \left( {{\varvec{\upalpha \upvarepsilon }}} \right)} \right]$$

where ${\varvec{\upsigma}}$ is the total stress tensor, ${\mathbf{M}}$ is the stiffness tensor of the drained porous matrix, ${\varvec{\upvarepsilon}}$ is the total strain tensor, ${\varvec{\upalpha}}$ is the Biot’s tensor, p is the pore pressure, $M$ is the Biot’s modulus, $\xi$ is the variations in fluid content, and $\text{tr}()$ is the trace operator.

The equilibrium equation:

3 $$\rho {\ddot{\mathbf{u}}}^{s} - \nabla \cdot {\varvec{\upsigma}} = {\mathbf{0}}$$

here, the total density $\rho$ is related to the porosity $\phi$, the density of solid phase $\rho_{\text{s}}$, and the density of fluid phase $\rho_{\text{f}}$, and the $\rho$ can be defined by relation $\rho = \phi \rho_{\text{f}} + (1 - \phi )\rho_{\text{s}}$, and ${\ddot{\mathbf{u}}}^{s}$ is the second derivative of the displacement.

Fluid mass conservation equation:

4 $$\frac{\partial \xi }{\partial t} = - \nabla \cdot {\mathbf{V}}$$

Darcy’s law:

5 $${\mathbf{V}} = - {\mathbf{k}}\left( {\nabla p + \rho_{\text{f}} {\ddot{\mathbf{u}}}^{s} } \right)$$

here, ${\mathbf{V}}$ is the velocity vector and ${\mathbf{k}}$ is the intrinsic permeability tensor which can be defined by the relation ${\mathbf{K}}$ = ${\mathbf{k}}\text{/}u$, where ${\mathbf{K}}$ is the permeability tensor and $u$ is the dynamic viscosity of interstitial fluid.

Inertia items in Eq. (3) and (5) can be ignored, because the bone is always subjected to low frequency cyclic loading in daily life and it has little effect on computing results. The simplified governing Eq. (6) is obtained by plugging (1) into (3) and plugging (2) and (5) into (4):

6 $$\left. \begin{aligned} &{\varvec{\upalpha}}\nabla p = \nabla \cdot \left( {{\mathbf{M\varepsilon }}} \right) \hfill \\ &\frac{1}{M}\frac{\partial }{\partial t}p - \nabla \cdot \left( {{\mathbf{k}}\nabla p} \right) = - \frac{\partial }{\partial t}\left[ {\text{tr} \left( {{\varvec{\upalpha \upvarepsilon }}} \right)} \right] \hfill \\ \end{aligned} \right\}$$

### Establishment of macroscale poroelastic mathematical model and FE model

In macroscale model, we neglected the bone marrow cavity and trabecular bone and established a hollow bone model in the cylindrical coordinate system (*r*, *φ*, *z*). As shown in Fig. 1A, *a*, *b,* and *h* represent inner radius, outer radius, and height, respectively. The surface of periosteum was set no-flow conditions [32]. Equation (7), reported by literature [33, 34], models a modified physiological arterial pressure pulse (in mmHg) as the hydrostatic pressure for the surface of endosteum.

7 $${P}_{0} = 8 0+ \frac{ 1 2 0- 8 0}{ 2} \times \left\{ \begin{aligned} & 0. 5+ 0. 5 {\text{cos}}(10\pi (t - 0.1)),0 < t \le 0.1 \hfill \\ & 1.5 - 0.5\cos (10\pi (t - 0.5)),0.1 < t \le 0.3 \hfill \\ & 0.5 + 0.5\cos ({5 \mathord{\left/ {\vphantom {5 {3\pi ({\text{t}} - 0.3)}}} \right. \kern-0pt} {3\pi ({\text{t}} - 0.3)}}),0.3 < t \le 0.9 \hfill \\ \end{aligned} \right\}$$

The cyclic loads were axial to represent longitudinal compression on the top and bottom surface [32, 35]. The solution of fluid pressure can be obtained:

8 $$p(r,t) = \frac{{\frac{{ME_{r} \left( {E_{z} - E_{r} v_{z}^{2} } \right)}}{{\left( {1 + \nu_{r} } \right)\left( {E_{z} - E_{z} v_{r} - 2E_{r} v_{z}^{2} } \right)}}\left( {\alpha c + \alpha \varepsilon_{z0} } \right)}}{{\frac{{E_{\text{r}} \left( {E_{z} - E_{r} v_{z}^{2} } \right)}}{{\left( {1 + \nu_{r} } \right)\left( {E_{z} - E_{z} v_{r} - 2E_{r} v_{z}^{2} } \right)}} + M\alpha^{2} }} \times \left[ {\frac{{I_{0} \left( {Cr} \right)K_{1} \left( {Cb} \right) + K_{0} \left( {Cr} \right)I_{1} \left( {Cb} \right)}}{{I_{0} \left( {Ca} \right)K_{1} \left( {Cb} \right) + I_{1} \left( {Cb} \right)K_{0} \left( {Ca} \right)}} - 1} \right]e^{i\omega t}$$

where *I*_*n*_ and *K*_*n*_ represent the first kind and the second kind modified Bessel function of order *n* (*n* = 0, 1), respectively. *ε*_*z*0_ is amplitude of axial strain, *E* is drained Young’s modulus (Pa), and *ν* is Poisson’s ratio of the drained porous matrix. $\alpha$ is the Biot-Willis coefficient, and c represents the integral constant determined by the boundary conditions. The *E*_*r*_, *ν*_*r*_ and *E*_*z*_, *ν*_*z*_ represent radial and axial drained Young’s modulus and Poisson’s ratio, respectively. The constant *C* can be determined by the relation:

9 $$C = \sqrt {i\omega \mu \left( {\frac{{E_{\text{r}} \left( {E_{z} - E_{r} v_{z}^{2} } \right)}}{{\left( {1 + \nu_{r} } \right)\left( {E_{z} - E_{z} v_{r} - 2E_{r} v_{z}^{2} } \right)}} + M\alpha^{2} } \right){{} \mathord{\left/ {\vphantom {{} {\frac{{kME_{\text{r}} \left( {E_{z} - E_{r} v_{z}^{2} } \right)}}{{\left( {1 + \nu_{r} } \right)\left( {E_{z} - E_{z} v_{r} - 2E_{r} v_{z}^{2} } \right)}}}}} \right. \kern-0pt} {\frac{{kME_{\text{r}} \left( {E_{z} - E_{r} v_{z}^{2} } \right)}}{{\left( {1 + \nu_{r} } \right)\left( {E_{z} - E_{z} v_{r} - 2E_{r} v_{z}^{2} } \right)}}}}}$$

where $i = \sqrt { - 1}$, $\omega$ represents load frequency, and *k* is intrinsic permeability.The fluid velocity *V* can be given by Darcy’s Law:

10 $$\text{V} = - \frac{k}{\mu }\frac{\partial p}{\partial r}$$

In order to provide the basis for researching the stress and strain field and fluid flow in various functional units by poroelastic FE method, the validity of poroelastic FE model was validated by comparing numerical result with simulation results. The analytical solution was obtained by MATLAB software. The poroelastic FE model was established by COMSOL Multiphysics. To simulate the mechanical environment of bone tissue, the load–displacement *w* of *z* direction was axial to represent longitudinal compression on the top and bottom surface. The amplitude of harmonic displacement (*w*) and frequency (*f*) is 0.5 μm and 1 Hz, respectively. The maximum axial strain ($\varepsilon$) is 1000 $\mu \varepsilon$ in a cycle:

11 $$w = A\left[ {\cos (2\pi ft) - 1} \right]\;[{\text{mm}}]$$

There are two kinds of porosities associated with bone fluid: the vascular porosity (order 20 μm) and the lacunar–canalicular porosity (order 0.1 μm). The range of permeability (*k*_vp_ and *k*_lcp_) that describes the fluid flow in the vascular porosity ($\phi_{\text{v}}$) and the lacunar–canalicular porosity ($\phi_{\text{lc}}$) are 10^−13^m^2^–10^−17^m^2^ and 10^−17^m^2^–10^−25^m^2^, respectively [28]. We set *k*_vp_= 10^−15^m^2^ and porosity $\phi_{\text{v}}$ = 0.04 to describe fluid flow in the vascular porosity and *k*_lcp_= 10^−19^m^2^ and porosity $\phi_{\text{lc}}$ = 0.05 to describe fluid flow in the lacunar–canalicular porosity. Croker et al. found that the cross-sectional diameter and thickness of cortical bone of human femur (22.1–32.8 mm) were thicker than animals by analysis and comparison of human, sheep, and kangaroos using statistical methods [36]. In order to simplify calculation and facilitate comparison of the upcoming animal experiment, the cross-sectional diameter and the height of segment bone are set 1 cm and 1 mm, respectively. The related parameters are shown in Table 1 [14, 27, 37].

### Macro–mesoscopic FE model

#### Establishment of macro–mesoscopic FE model

It is difficult to study the relationship of fluid flow within different levels and functional units by experimental and theoretical methods; therefore, the FE simulation method becomes the first choice. As shown in Fig. 1B, the macro–mesoscopic model considered the endosteum, osteon, interstitial bone, and periosteum, but the Haversian canal and the marrow cavity in bone were neglected (Fig. 1C). As shown in Fig. 1d, due to symmetry, only 1/8 model was observed in the computations. The radius of osteon was set *R*_o_ = 150 μm, and the Haversian canal radius is set *r*_o_ = 50 μm. The periosteum is a strong connective tissue envelope covering the bone surface except joints. It was tightly bound to the outer wall of bone tissue, and the thickness was set 150 μm [38]. The endosteum was a thin connective tissue envelope covering the bone marrow cavity and bone trabecula, and the thickness was set 50 μm.

#### Boundary conditions and material parameters

The surface of periosteum and bone marrow were set to the same value as the macroscopic model. The FP of Haversian canal was always ignored in previous study [10, 14, 27]; however, the span of both *k*_vp_ and *k*_lcp_ was very large [28], and when the gap of permeability value between *k*_vp_ and *k*_lcp_ was not that big, the FP of Haversian canal couldn’t be ignored. In macro–mesoscopic model, the FP of Haversian canal was derived from the calculation results of the corresponding locations of macroscale model. The load–displacement is the same as macroscale model.

Due to the symmetry of full model, the both sides and bottom surface of the segment 1/8 model which cut from the whole model applied constrained symmetrically to prevent rigid body motion. Because the endosteum, osteon, and interstitial bone and periosteum were tightly bound together, all geometric objects were tied together by using the function of Form Union in COMSOL Multiphysics software to make the condition of the mesh border lines continuous.

The Young’s modulus (*E*) of interstitial bone was 10% larger than osteon, and the Poisson ratio (ν) was 10% smaller than osteon [39]. The transverse isotropic elastic constants for cortical bone were used. McBride et al. measured that the axial and radial elastic modulus of periosteum were 18.8–32.5 MPa and 3.19–5.62 MPa [40], respectively, and the average values 25.65 MPa and 4.41 MPa were used in this model. The Poisson’s ratio was 0.49 [19]. The permeability of the periosteum from the bone to muscle surface of periosteum and the muscle to bone surface of the periosteum was much different, and we only considered the permeability (2.7 × 10^−16^ m^2^) between the periosteum and the bone tissue [41]. For other poroelastic material parameters, we set the same value on macroscopic and macro–mesoscopic model. The material parameters used in the FE model are shown in Table 2 [14, 19, 37, 40, 41].

Different precision of meshes were studied, to demonstrate that the results were converged with respect to mesh refinement—any further refinement of the mesh would only marginally improve the precision of the results. A Free Tetrahedral mesh was used and included 48,375 elements.

### Mesoscopic FE model

#### Establishment of FE mesoscale model including macro–meso interface

The establishment of mesoscale model consisted of two steps. Firstly, the macroscopic and macro–mesoscopic models were analyzed in order to capture the general trends and to identify the critical part. Secondly, a fine mesoscale model containing the critical part was made, and the study was resolved. In order to allow for smooth transition between macroscale and mesoscale, the FE mesoscopic model including macro–meso interface was established (Fig. 1g). The change of material parameters was consistent with the mesoscale model.

#### Establishment of mesoscopic FE model

The osteon was a basic unit for organizing and constructing the cortical bone, which composes of multilayered quasi-cylindrical composites of compact tissue arranged in 7–10-µm-thick lamella around the Haversian canal (Fig. 1i) [20]. Each osteon lamella had different properties [26]. Each osteon was encircled in a 1–5-μm-thick interface structure form, which was called the cement line, to separate osteon from interstitial bone (Fig. 1j). In the analysis, 10 bone lamellae were established. The cement line was set 1 µm [42]. The global effects from the macroscale model were transferred to the mesoscale model via appropriate boundary conditions. By the analysis of the simulation results of macroscale model, we confirmed the critical part near the outer region of periosteum as the position of mesoscale model. As shown in Fig. 1h, the mesoscale model was a cube (340 µm) that cut from the macro–mesoscopic model near the periosteum, which contained one osteon.

#### Boundary conditions and material parameters of the mesoscale model

The mesoscale model was based on the coupling of Structural Materials Module and Darcy’s law in COMSOL Multiphysics. Since the model contained the properties of structural mechanics and fluid flow behaviors that were modeled with a poroelastic material, many degrees of freedom were required in order to obtain the accurate behaviors of poroelastic mechanics in all functional units. The concept of this technique required that an analysis of the macro–mesoscopic model was performed in order to capture general trends, followed by an analysis of a mesoscale model which the macro–mesoscopic structure units subdivided into mesoscale structure units were studied in detail. The result of the macro–mesoscale, the displacement and pore pressure, were prescribed directly to the boundaries where the mesoscale model was cut out of the macro–mesoscale and via strain and pressure gradient transfer to the mesoscale model, which ensured the continuity of the two scales.

Each lamella was given different value of *E* and *k*_lcp_. *E*_*r*_1, and *E*_*z*_1 represent the radial and the longitudinal Young’s modulus of lamella 1 (Fig. 1i), respectively, *E*_*r*_2, and *E*_*z*_2 represent the radial and the longitudinal Young’s modulus of lamella 2 (Fig. 1i), respectively, and so on. Generally speaking, the distribution of *E* along radial direction could be divided into the following three cases: Case 1: The value was high in the middle bone lamellae and low in lateral and medial bone lamellae [20]. Case 2: There was an increasing trend of *E* from the medial to the lateral [21]. Case 3: There was a decreasing trend of *E* from the medial to the lateral [22, 23].

As same as Chen et al., we assumed the osteon lamellae were all perfectly bonded, and each lamella had different permeability [10]. *k*_lcp_1 represents the lacunar–canalicular permeability of lamellae 1 (Fig. 1i), and *k*_lcp_2 represents the lacunar–canalicular permeability of lamellae 2, and so on. Three cases of permeability were considered in this model. In Case 4, *k*_lcp_ decreased linearly from lamella 1 to lamella 10. In Case 5, *k*_lcp_ increased linearly from lamella 1 to lamella 10, and in Case 6, the *k*_lcp_ had a symmetric distribution. The reference model was the macro–mesoscopic model which *E* and *k*_lcp_ were a fixed value at the corresponding position. The specific parameters of *E* and *k*_lcp_ in each lamella were shown in Tables 3 and 4.

## Data Availability

Data are available from the corresponding author upon reasonable request.

## Abbreviations

FP: fluid pressure
FV: fluid velocity
VMS: von Mises stress
MPS: maximum principal strain
*E*: Young’s modulus
*k*_lcp_: lacunar–canalicular permeability
*k*_vp_: vascular permeability
$\phi_{\text{v}}$: lacunar–canalicular porosity
$\phi_{\text{lc}}$: lacunar–canalicular porosity
